# The Harmful Footprint of Aged Biomicroplastics on Algal Development: A Comparative Study of Polylactic Acid, Polyhydroxybutyrate, and Cellulose Acetate

**DOI:** 10.1021/acsomega.5c06359

**Published:** 2025-10-29

**Authors:** Paula Walz, Simon B. Redlich, Marius Hermesdorf, Laura Calderón-Rodríguez, Marcus Franke, Desirée Leistenschneider, Quirina Roode-Gutzmer, Felix H. Schacher, Michael Stelter, Thomas Wichard, Patrick Braeutigam

**Affiliations:** † 489063 Friedrich Schiller University Jena Faculty of Chemistry and Earth Sciences, Institute of Technical Chemistry and Environmental Chemistry, Philosophenweg 7a, Jena, Deutschland 07743, Germany; ‡ 9378 Friedrich Schiller University Jena Faculty of Chemistry and Earth Sciences, Institute of Inorganic and Analytical Chemistry, Lessing Str. 8, Jena, Deutschland 07743, Germany; § Friedrich Schiller University Jena Faculty of Chemistry and Earth Sciences, Institute for Organic Chemistry and Macromolecular Chemistry, Lessing Str. 8, Jena, Deutschland 07743, Germany; ∥ Friedrich Schiller University Jena Center for Energy and Environmental Chemistry Jena, Philosophenweg 7a, Jena, Deutschland 07743, Germany; ⊥ Fraunhofer IKTS Hermsdorf, Michael-Faraday-Str. 1, Hermsdorf, Deutschland 07629, Germany; # University of Stuttgart Faculty 2 Civil and Environmental Engineering, ISWA, Institute for Sanitary Engineering, Water Quality and Solid Waste Management, Bandtäle 2, Stuttgart, Deutschland 70569, Germany; ∇ University of Stuttgart Faculty 2 Civil and Environmental Engineering, Micropollutants Competence Centre Baden-Württemberg, Bandtäle 2, Stuttgart, Deutschland 70569, Germany

## Abstract

Biopolymers are increasingly produced as sustainable alternatives to plastics, but their degradation in aquatic ecosystems raises ecological concerns. This study demonstrates that the photodegradation of polylactic acid (PLA), polyhydroxybutyrate (PHB), and cellulose acetate (CA) in artificial seawater substantially increased toxicity under the elevated laboratory conditions used, compared with virgin counterparts, adversely affecting the development and growth of seaweeds. Those aged biopolymers and their leached substances impaired the growth and development of the green macroalgae *Ulva* (Chlorophyta) under standardized conditions, but the ecological relevance at natural seawater concentrations is likely lower. Chlorophyll *a* fluorescence, median lethal concentration (LC_50_-values), and HR-MS analysis corroborated these findings, emphasizing detrimental impacts on the fitness and development of *Ulva mutabilis*. Toxic effects were linked to substances released during photodegradation and hydrolysis, especially for PLA and PHB. PLA exhibited a 6.7-fold increase in toxicity following UV exposure; LC_50_-values indicate increased toxicity from direct polymer exposure and from leached compounds at the applied concentration. PHB exhibited the strongest degradation-related toxicity, with over a 10-fold increase. CA was the most toxic overall, with an LC_50_-value of <0.5 mg/mL in aged samples under laboratory conditions. Even nonaged CA showed effects at these elevated concentrations, but environmental relevance remains uncertain. Although this is an explorative model study in lab scale using elevated concentrations, the observed persistence and (post-)­degradation effects of biomicroplastics highlight potential polymer-specific toxicity and emphasize the need for further research on the degradation of biopolymers in the marine environment.

## Graphical abstract

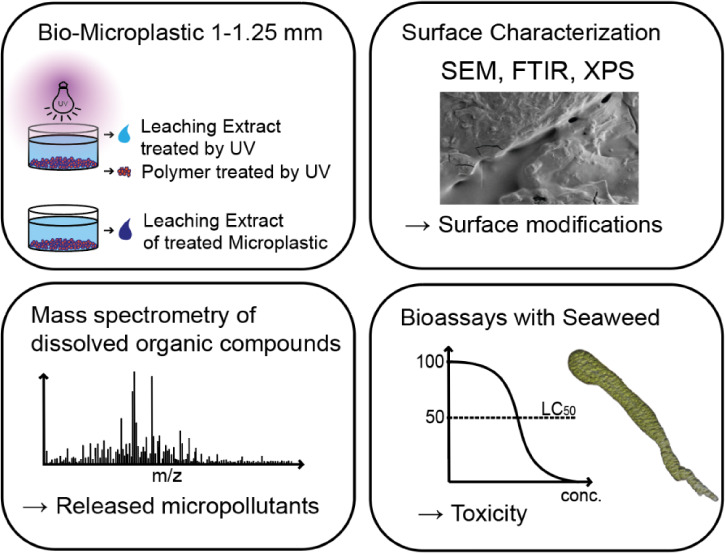

## 1 Introduction

Polymer production has risen significantly in recent years,^1,2^ reaching a record of 413.8 million tons in 2023.^3^ Plastic enters the oceans mainly via land^4^ and can now be found in many environmental compartments.^5,6^ This results in microplastic accumulation, formed through photochemical, mechanical degradation, and long-term mineralization processes over years or decades,^7^ often changing material properties and increasing toxicity or environmental effects.^8–10^

Exposure to environmental factors, such as ultraviolet (UV) radiation, temperature fluctuations, or chemical agents, can degrade the polymer structure, leading to cracks, fractures, or increased surface roughness.^9,11^ This can facilitate the release of chemical compounds, e.g., additives or other components contained in the polymers.^12,13^ Furthermore, aging can result in the formation of potentially toxic degradation-related compounds^8^ that may leach into the aquatic environment.

The growing production of biopolymers,^3^ such as PLA, PHB, and CA, is often presented as a sustainable alternative to conventional plastics, as they are derived from renewable resources and promoted as “environmentally friendly” precisely by virtue of their faster degradability.^14,15^ However, biopolymers labeled as “biodegradable” often do not degrade under environmental conditions (e.g., temperature, microorganism concentration, humidity).^16^

Biopolymers in the environment undergo aging and degradation processes, potentially releasing leached substances with unknown ecological effects. These substances may harm aquatic habitats, disrupt food webs, and pose long-term ecological consequences.^17^ The exact degradation dynamics in natural environments remain unclear. Degradability is mainly studied under controlled conditions like industrial composting facilities, rarely under home-composting or natural conditions, where biopolymer degradation often takes much longer.^18,19^ While natural degradation dynamics remain complex and not fully understood, laboratory-based studies using simplified model systems allow controlled investigation of toxicity and aging processes. Such approaches provide mechanistic insights under reproducible conditions, even though they do not fully capture the complexity of natural marine environments.

To address these challenges, it is essential to develop a better understanding of the aging mechanisms of biopolymers in the marine environment and analyze the potentially toxic effects of leached substances and degraded biomicroplastics themselves.

To assess the impact of pollutants arising from aged and nonaged biomicroplastics, the model organism *Ulva mutabilis* was chosen^20–22^ serving as an exploratory, laboratory-scale model study. *Ulva* is a globally widespread macroalga^23^ that plays a critical role in marine ecosystems by supporting energy flow, providing habitat, and sustaining numerous ecologically and economically important species.^24^

It serves as an ideal model organism for *in vitro* toxicity tests, as all life stages can be studied from settling up to adult, mature life stages under laboratory conditions.^25,26^ Throughout its life cycle, the algae must cope with natural adverse effects. The effects of degraded polymers and their leached substances on the algae can be assessed through changes in organismal size, physiological stress responses, chlorophyll fluorescence, and survival rates.

In this study, aged and nonaged biomicroplastics were compared using surface characterization methods. Specifically, we aimed to address two key questions: (i) how aging and UV-induced degradation affect the toxicity of PLA, PHB, and CA microplastics and their leached substances toward early life stages of the macroalga *U. mutabilis* and (ii) whether toxic substances continue to be released after exposure ends. The toxicity of the biomicroplastics and their associated leached substances during and after aging was assessed using bioassays with the alga *U. mutabilis*, providing a macroalgal model suitable for investigating gametes and gametophytes under controlled laboratory conditions. To ensure reliable determination of LC_50_ values, PLA and PHB were tested at concentrations up to 250 mg/mL, while CA was tested up to 20 mg/mL due to its higher toxicity. In addition, the chemical composition of the leached substances was analyzed.

## 2 Results and Discussion

### 2.1 Surface Characterization of Aged and Nonaged Biomicroplastics

Using Fourier-transform infrared spectroscopy (FTIR), X-ray photoelectron spectroscopy (XPS), and scanning electron microscopy (SEM), the surfaces of the three irradiated and nonirradiated biomicroplastics were compared. As expected, PLA showed the strongest aging effects, followed by PHB, while CA remained unchanged. In the XPS measurement (see Section S-3.2), PLA showed the strongest aging effects, as evidenced by an increased oxygen content from 12.6 at% to 34.9 at% and significant changes in the C 1s spectrum of PLA_aged_. Due to the aging, peaks in the higher binding energy region of the C 1s spectrum evolved, which was due to strong surface oxidation and the beginning of the decomposition/modification of the molecular structure by the breaking of hydrocarbon chains.^27–29^ PHB and CA in particular showed only minor changes compared to PLA. In PHB, the oxygen content was slightly higher, and the peak in the C 1s at 289.1 eV increased after aging, indicating the presence of COOH groups. In the C 1s spectrum of CA_aged_, the peak at 286.9 eV was the most intense, which points to the formation of OH groups on the surface during aging. The SEM images confirm these results: PLA_aged_ developed pronounced cracks, PHB_aged_ showed scattered cracks, and CA_aged_ showed no cracks. Surface changes, such as increased roughness and cracking due to weathering, are classic features of polymer aging.^11,30^ FTIR analysis showed no significant photoaging effects besides the expected polymer spectra.^31,32^ For PHB, our FTIR results correspond to the literature;^30,33^ aging of PLA in the absence of water would result in detectable changes in the surface functional groups,^30,31^ but these are not visible with artificial seawater. CA has an absorption maximum at 260 nm and ages only slowly in sunlight (>300 nm).^34,35^ The lamp used emitted light between 250 and 450 nm, but with low intensity below 350 nm, so that no significant photodegradation occurred after 350 h,^36^ which was confirmed by the analyses.

### 2.2 Effect of Aged and Nonaged Microplastic Particles on the Development of *Ulva*

The declining survival rate of *Ulva* germlings exposed to PLA_aged_ and PLA_virgin_ indicated the inherent toxicity of microplastics, which was exacerbated by UV-induced aging. PLA_virgin_ exhibited an LC_50_ value of 158.03 mg/mL, while PLA_aged_ showed a 4.5-fold increase in toxicity, with an LC_50_ of 35.08 mg/mL (Figure 1a). The increased toxicity of degraded PLA might result from (1) a higher amount of leached compounds during the cultivation phase of the bioassay, (2) reduced particle size and cracking (a larger surface area may also increase leaching), or (3) modification of PLA surface functional groups.^37^

**Figure 1:**
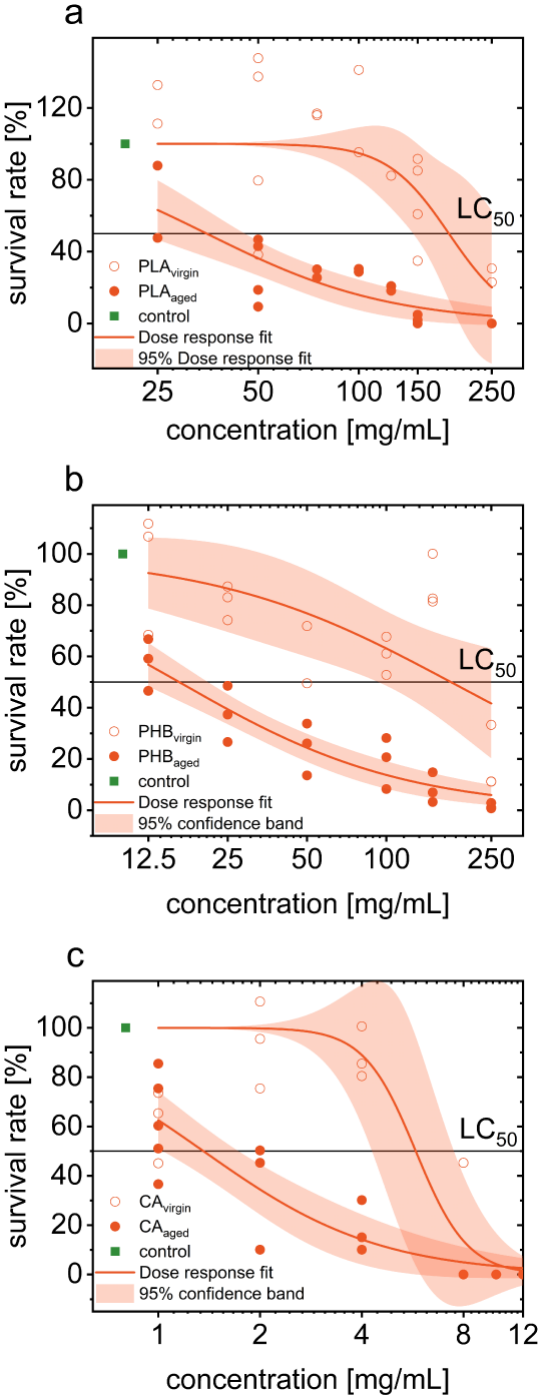
Bioassay results showing the toxic effects of aged and virgin (a) PLA, (b) PHB, and (c) CA biobased microplastics on *U. mutabilis* gametes (density: ∼2 gametes/μL). Survival rates are obtained from concentration–response curves, including LC_50_ values and 95% confidence bands. The pH ranged from 7.88 to 8.22 (SD ± 0.09).

The adverse effects of untreated PLA nanoplastics have already been demonstrated in *Hydra viridissima* and *Danio rerio* at relatively low concentrations (100 mg/L), resulting in malformations, disrupted physiological processes, and reduced larval locomotor activity.^32^ The impact of these polymers is strongly influenced by particle size.^38^ However, in this study, microplastics (1–1.25 mm) were used instead of nanoplastics. Notably, several studies have confirmed increased toxicity of PLA following UV irradiation (in water or air), reporting the formation of free radicals, polymer chain scission, and oxidative degradation products.^39,40^

PHB showed the highest aging-related toxicity measured in this study. It increased by a factor of 10.7. The LC_50_-value of PHB_aged_ was 16.35 mg/mL, and that of PHB_virgin_ was 175.49 mg/mL (Figure 1b). Nevertheless, there is a basic toxicity of nonaged PHB microplastics similar to PLA_virgin_.

There is no consensus about the toxic effect of PHB nanoparticles. They are considered nontoxic (study on *Artemia franciscana*),^41^ or exhibit low to high toxicity (study on *Hydra viridissima*, *Lates calcarifer*), with effects such as altered feeding behavior or tissue translocation, but not mortality (LC_50_).^42,43^ The concentrations used in these studies were lower than those in this study, and they focused on nanoplastics. The larger surface-to-volume ratio of nanoplastics can increase reactivity, promoting the release of harmful substances and interaction with cells.^38,44^

The LC_50_-value of CA_aged_ (1.35 mg/mL) was significantly lower than that of CA_virgin_ (5.77 mg/mL) (Figure 1c), which indicates a higher toxicity. CA is thus more toxic than PLA and PHB. As the main component of cigarette filters, it is one of the world’s most problematic wastes—around 4.5 trillion pieces are disposed of every year. Due to its slow decomposition and high toxicity when aged, CA jeopardizes marine ecosystems.^45^

The three polymer types exhibit two forms of toxicity: an inherent baseline toxicity from the untreated materials and an enhanced age-related toxicity following photochemical degradation. While the exact mechanisms remain unclear, previous studies suggest that the formation of reactive oxygen species (ROS) may induce oxidative stress in exposed organisms.^8^ This is supported by XPS analysis, which revealed an increased oxygen content on the surface of PLA (to a lesser extent on PHB), indicating the formation of stable oxygen-containing functional groups such as carbonyls, peroxides, and hydroxyls, although this was not explicitly investigated.

The survival rate revealed that all UV-irradiated polymer samples had a significantly greater negative impact on the development of gametes and juvenile gametophytes of *U. mutabilis* compared to the control. Notably, this effect was observed despite minimal structural changes in the polymers, as confirmed by SEM and FTIR analyses (Figures S2 and S6). These findings highlight the disconnect between physical integrity and biological impact. As a result, the chemical fingerprint (i.e., dissolved organic matter) of the aged biopolymers was further investigated, with a particular focus on cellulose acetate.

### 2.3 Release of Leached Compounds by Aging Biopolymers

Leaching Extracts_1 and 2 from both aged and nonaged (virgin) polymer samples were analyzed using high-resolution mass spectrometry (HR-MS). A blank sample consisting of the untreated *ulva* culture medium (UCM) without polymer residues was also analyzed and subtracted from all data sets to account for background signals. These analyses focused on cellulose acetate (CA), as this polymer exhibited the highest toxic effects in comparison to the other polymers tested (PLA and PHB).

The hierarchical clustering (Figure 2b) yielded a meaningful subdivision into eight terminal leaves across four main branches, corresponding to the four sample types analyzed in duplicate (Figure 3b). The high precision of the cluster assignment demonstrates the effectiveness of the applied clustering approach in differentiating sample origins. The distinct separation of sample groups highlights the robustness of the method and underscores its utility for the reliable classification of polymer-derived leaching extracts in environmental assessments.

**Figure 2:**
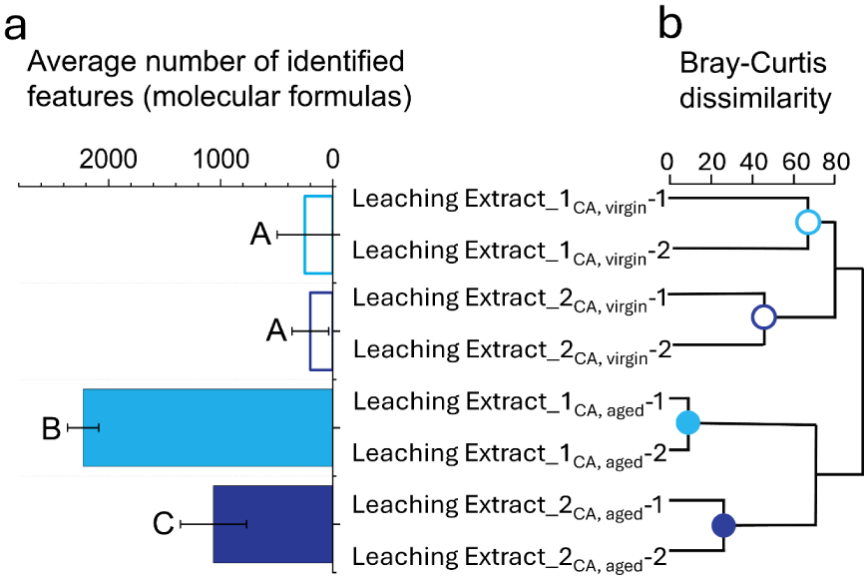
Analysis of the dissolved organic matter released by aging biopolymers. (a) Average number of identified molecular formulas with statistical groupings based on significant differences (*p* < 0.05) (Welch-ANOVA), and (b) dendrogram of Leaching Extract_1 and 2 (aged and virgin) in replicas. UCM was extracted as a blank.

**Figure 3:**
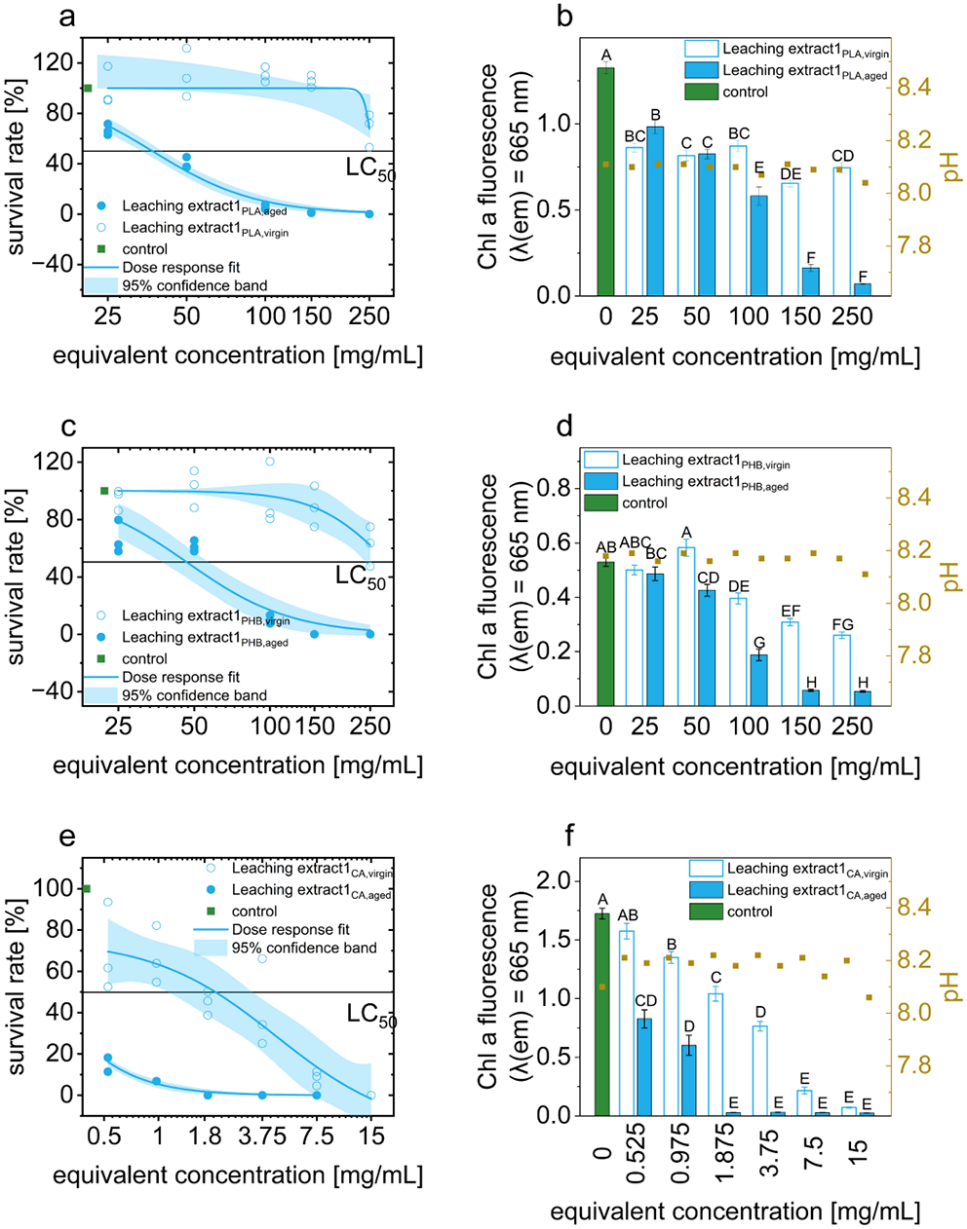
Toxic effects of aged and virgin Leaching Extracts_1 obtained from (a,b) PLA, (c,d) PHB, and (e,f) CA microplastics on the germination of *U. mutabilis* gametes (density: ∼2 gametes/μL). Results included survival rates shown as concentration–response curves with LC_50_ values and 95% confidence bands, chlorophyll *a* fluorescence with standard error, and statistical groupings based on significant differences (Dunn–Šidák test). The pH values were also recorded.

A comparison of the number of molecular formulas (Figure 2a) identified across the four samples—comprising over 2500 distinct features—revealed a clear separation into subclusters. The negative control exhibited the lowest number of detected features, reflecting the minimal presence of relevant molecular compounds. Both virgin samples (Leaching Extract_1_CA,virgin_ and Leaching Extract_2_CA,virgin_) contained 250 and 202 molecular formulas on average, indicating a relatively low chemical diversity. In contrast, the aged sample Leaching Extract_1_CA,aged_ displayed the highest molecular complexity, with 2226 distinct formulas detected, strongly suggesting the release of a broad range of substances due to photochemical degradation. The aged Leaching Extract_2_CA,aged_ sample showed an intermediate profile, with 1065 molecular formulas identified, significantly higher than in the untreated samples, further confirming the enhanced leaching of degradation products following UV exposure.

### 2.4 Leaching of Substances during Photodegradation of Biomicroplastics (Leaching Extract_1)

After showing in the previous section that UV radiation on microplastics releases leaching substances into the medium, the following section investigates the influence of these substances on *U. mutabilis* gametes. The polymers degraded in this study have a density of >1 g/cm^3^, which was deliberately chosen so that the polymers are irradiated through a 3.5 cm water column, and the Leaching Extract_1 was created, which was used for the subsequent experiments.

UV irradiation releases toxic substances from PLA microplastics into the medium, as shown by the LC_50_-values of Leaching Extract_1_PLA,aged_ at 37.04 mg/mL and Leaching Extract_1_PLA,virgin_ at over 250 mg/mL (Figure 3a), which corresponds to a 6.7-fold increase. The chlorophyll *a* fluorescence shows a significant effect even at the lowest equivalent concentration tested (25 mg/mL) (Figure 3b).

These toxic effects might potentially be explained by the combination of hydrolysis and photodegradation, during which micro- and nanoparticles are released,^27^ which then form toxic PLA oligomers and monomers in water.^46^ This occurs through the decomposition of ester bonds^46^ as well as processes such as photoionization and polymer chain scission^29,47^ that occur during photolysis.

Previous studies have already found a low toxicity of PLA-leached substances but without prior UV treatment.^17,48^ Other studies follow similar explanatory approaches, but our focus is on the interaction between photodegradation and hydrolysis.

Similar to PLA, PHB also exhibits aging-related leaching (Figure 3c,d), indicating the release of toxic substances into artificial seawater during the photodegradation process. In the absence of UV-induced aging, only minor effects were observed, with no LC_50_-value reached within the tested concentration range, suggesting it exceeds 250 mg/mL. In contrast, the toxicity of Leaching Extract_1_PHB,aged_ was significantly higher, with an LC_50_ of 46.95 mg/mL, which is approximately 5.3 times lower than that of its virgin counterpart. Fluorescence measurements further supported these observations, showing detectable stress responses in *Ulva* at 50 mg/mL for Leaching Extract_1_PHB, aged_ and at 100 mg/mL for the virgin extract. These concentrations were approximately two and four times higher, respectively, than those required for comparable effects with PLA leaching extracts. This indicated that the degradation products of PHB exerted a less pronounced impact on algal stress levels compared to those released from PLA.

In contrast to our study, a study on leached substances derived from PLA and PHB-*co*-hydroxyvalerate (comparable to PHB in terms of their degradation products) found a higher toxicity for PHB-*co*-hydroxyvalerate, though different test organisms and lower microplastic concentrations were used.^17^ Similarly, another study investigating PHB resin, PLA, and PLA/PHA using larvae of *Paracentrotus lividus* also identified PHB as the most toxic material.^49^

It is hypothesized that the observed increase in the toxicity of Leaching extract_1_PHB,virgin_ may result from the formation of free radicals, which are generated through Norrish Type I reactions that abstract hydrogen atoms and induce polymer chain scission, leading to the formation of small, potentially water-soluble polymer fragments and carbonyl-containing degradation products.^30,50^ The resulting ROS could lead to progressive oxidative damage and thus^51,52^ to increased mortality and an increased stress level of the gametes and gametophytes.

An LC_50_-value of 1.62 mg/mL was measured for the untreated Leaching Extract_1 sample of CA, while the LC_50_-value for the photodegraded sample was below the lowest equivalent concentration measured (<0.5 mg/mL). These are the most toxic values measured in a sample in this study (Figure 3e). The fluorescence measurements confirm the toxicity assessment of the survival rate. The Leaching Extract_1_CA,aged_ already deviates at the lowest equivalent concentration (0.525 mg/mL). The Leaching Extract_1_CA,virgin_ deviates significantly from the control sample at the second equivalent concentration (0.975 mg/mL) (Figure 3f).

These results were consistent with mass spectrometric analysis. Most molecular formulas (*N* = 2226) were identified in the Leaching Extract_1_CA, aged_ (Figure 2), which may result in higher toxicity. Irradiation either enhanced the release of existing substances from CA or favored the formation of toxic degradation products that subsequently leached into the surrounding medium. As previously reported, irradiation of CA in water leads to the release of compounds such as acetic acid,^53^ while also generating methane, carbon monoxide, and carbon dioxide, all of which can disrupt the chemical stability of the material.^35^ Since the acetic acid could not have had a negative effect on the gametes of the algae due to the buffer used during the bioassays, it seems plausible that other, as yet unidentified photochemical degradation products, such as low-molecular aldehydes, ketones, or peroxides, could be responsible for the observed effects. To date, no studies have been found in the literature examining the irradiation of cellulose acetate (CA) and the composition of the resulting leaching extracts.

All three polymers exhibited an accelerated release of leached substances upon exposure to UV radiation. These leached substances were presumed to consist primarily of low-molecular-weight degradation products formed through polymer breakdown, as previously reported.^54^ While several studies have documented toxic effects associated with similar leachates, specific investigations on CA are lacking, and only limited research on PHB is available in the current literature.^55^ Direct comparisons across studies are challenging due to differences in experimental conditions, including model organisms, irradiation duration and intensity, plastic size, and polymer type. The observed variability in toxicity—both between irradiated and virgin samples—can likely be attributed to differences in chemical structure, functional groups, and degradation kinetics among the tested polymers.^39^

### 2.5 Effect of Leaching in the Postirradiation Phase (Leaching Extract_2)

After the effects of UV-induced leaching substances have been investigated, the following section examines whether previously irradiated polymers continue to release toxic leaching substances. The Leaching Extract_2 of the previously irradiated PLA (LC_50_ = 82.52 mg/mL) shows a 2.4-fold and 2.2-fold lower toxic effect than the aged PLA and the Leaching Extract_1_PLA,aged_ (Figure 4a). No toxic effect could be detected with Leaching Extract_2_PLA,virgin_ (LC_50_ > 250 mg/mL). Nevertheless, the untreated sample also has a toxic effect, as the chlorophyll *a* fluorescence (Figure 4b) shows significant deviations from that of the control sample at the lowest measured equivalent concentration (25 mg/mL). The Leaching Extract_2_PHB,virgin_ shows no effect on the survival of the gametes; the LC_50_-value is thus >250 mg/mL (Figure 4c), which corresponds to the highest measured equivalent concentration and thus the lowest PHB toxicity. Unlike the Leaching Extract_2_PHB,aged_, these have an LC_50_-value of 204.10 mg/mL. Even after the aging process has stopped, degraded PHB samples release leaching substances into the liquid medium. The results of the chlorophyll *a* fluorescence (Figure 4d) show a significant deviation at an equivalent concentration of 150 mg/mL, which confirms the survival rate results, as these also show a deviation before the LC_50_-value in previous experiments.

**Figure 4:**
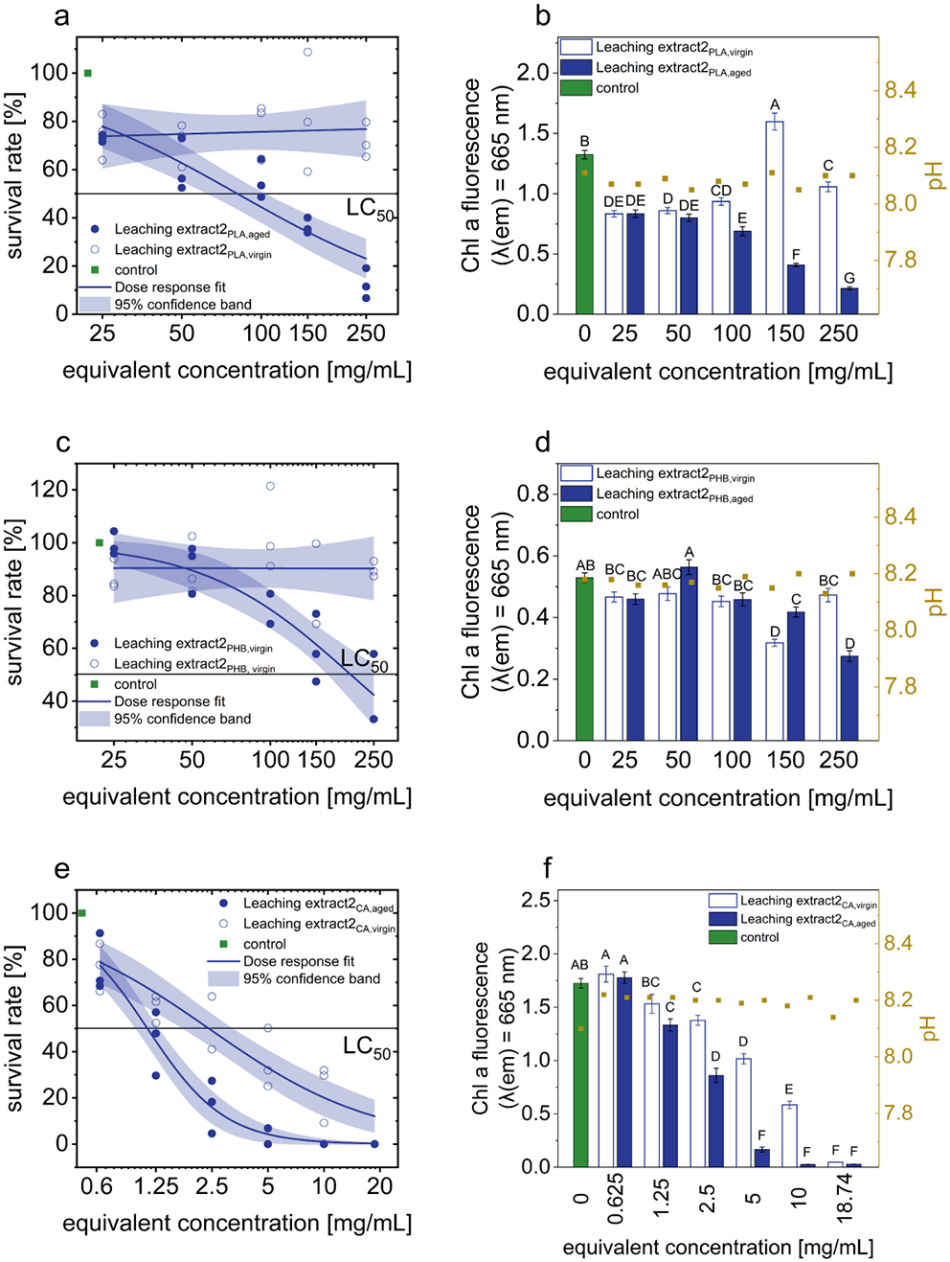
Toxic effects of aged and virgin Leaching Extracts_2 obtained from (a,b) PLA, (c,d) PHB, and (e,f) CA microplastics on the germination of *U. mutabilis* gametes (density: ∼2 gametes/μL). Results include survival rates shown as concentration–response curves with LC_50_ values and 95% confidence bands, chlorophyll *a* fluorescence with standard error, and statistical groupings based on significant differences (Dunn–Šidák test). The pH values were also recorded.

Similar to PLA, the toxic effect of PHB polymers can be attributed either to the surface of the plastic or to the leaching during the bioassay, i.e., after the aging process has stopped. The results of Leaching Extracts_2 provide a possible explanation for the negative effects on the gametes and juvenile gametophytes observed in the presence of the biomicroplastics. During the duration of the bioassay in the presence of the polymers (Figure 1) the Leaching Extracts_2 could leach into the medium of the gametes. Due to the experimental design of the bioassay, the gametes and juvenile gametophytes were not necessarily in direct contact with the polymers, so we concluded that a change in the surface of the polymers did not influence the algal development directly through microplastic-to-cell contact.

The Leaching Extract_2_CA,aged_ exhibited an LC_50_-value of 1.12 mg/mL, while Leaching Extract_2_CA,virgin_ showed an LC_50_-value of 2.41 mg/mL (Figure 4e). These results are consistent with the chlorophyll *a* fluorescence measurements, which revealed a significant difference in the irradiated sample at 1.25 mg/mL, whereas a comparable effect in the nonaged sample was observed only at a higher concentration of Leaching Extract_2_CA,virgin_ (Figure 4f).

As a semiquantitative technique, HR-MS analysis provided relative intensity values that generally correlated with compound concentration; however, due to factors such as ion suppression and the nature of the measurement principle, only approximate quantification is possible. The observed intensity values (Table S-6) followed the trend: Leaching Extract_1_CA,aged_ > Leaching Extract_2_CA,aged_ > Leaching Extract_1_CA,virgin_ > Leaching Extract_2_CA,virgin_. This pattern corresponded with the measured LC50-values (Figures 3e,f and 4e,f) and the number of identified molecular formulas, suggesting a relationship between UV exposure and changes in the chemical composition of the leachates.

Our study demonstrates that all three tested biopolymers continued to release toxic substances into the surrounding medium even after UV irradiation, likely as a result of photodegradation processes that broke down polymer chains and promoted the leaching of degradation products. Surface cracking observed postirradiation further increased the polymers’ surface area, which may enhance the release of these substances.^56^

## 3 Conclusion

Despite the growing interest in bioplastics, research on the leaching behavior and ecotoxicological effects of environmentally aged biopolymers remains limited. While previous studies have associated increased toxicity in plastic products with additives,^57^ direct comparisons are often hampered by the use of commercial formulations that contain multiple, often unidentified, compounds rather than pure polymer bases.

Our exploratory, laboratory-scale model study demonstrated that unaged PLA, PHB, and CA exhibit toxicity toward early life stages of *U. mutabilis* and potentially other marine organisms. This toxicity appears to arise both from the polymers themselves and from substances leached into the surrounding medium. Moreover, UV exposure was found to significantly enhance the toxicity of both the biobased microplastic particles and their leachates, with additive-free PHB showing the greatest increase in toxic potential.

Importantly, our findings also show that the release of toxic compounds does not cease immediately after UV irradiation is discontinued. Toxic substances continue to leach from the particles into the environment even after the exposure ends, affecting *Ulva mutabilis* gametes and gametophytes. These findings are limited to the laboratory conditions tested, the species studied, and the elevated concentrations used to determine LC_50_; as an exploratory, laboratory-scale model study, our results provide insights into potential toxic mechanisms but do not directly predict effects in natural marine communities. Moreover, it should be emphasized that these results apply only to the specific commercial grades of PLA, PHB, and CA tested; while the materials were assumed to be largely free of residual additives, differences in polymer composition, synthesis routes, or minor undetected compounds may influence biological outcomes.

These results underscore the need to critically reassess the environmental safety of bioplastics, particularly under realistic aging conditions. They further highlight the importance of implementing more rigorous testing and long-term impact assessments to ensure that bioplastics do not pose unforeseen risks to marine ecosystems.

## 4 Methods

### 4.1 Study Design

This study examines the impact of photochemical aging of biobased microplastics (PLA, PHB, CA) on the toxicity toward gametes and gametophytes of the alga *U. mutabilis*, with the hypothesis that aging may generate new or enhanced toxic effects. Upon entering the oceans, plastic particles undergo such photochemical aging and degradation.^5,9^

Different studies report varying microplastic concentrations: 0.002–62.50 pieces/m^3^ across all oceans,^58^ 1000–890,000 pieces/km^2^ in the North Pacific,^59^ 0–1.61 pieces/m^2^ in the Ionian Sea,^6^ and 8.9–29.3 particles/L in Osaka Bay.^60^ Data variability arises from nonstandardized sampling, ocean dynamics, and differing microplastic properties.^58^

The concentrations used in this study are higher than those under actual environmental conditions. However, such conditions could be found in hotspots such as ocean eddies, reservoirs, and beaches.^9^ In addition, PLA and PHA (including PHB) are increasingly being produced.^61^ The investigation under elevated concentrations allows a sensitive detection of potential effects; if no effects occur even under these conditions, environmental risks at realistic exposures are to be classified as low.^62^ Biomicroplastics (PLA, PHB, CA) were UV-aged in artificial seawater to simulate ocean conditions; this is the native medium of *U. mutabilis* used in the bioassay. The leaching extracts formed during aging (called **Leaching Extract_1**) and the leaching extracts of already aged biomicroplastic particles (substances after irradiation (**Leaching Extract_2)**) were analyzed and compared to nonaged control samples based on algal development. Effects were assessed via juvenile gametophyte survival rates (LC_50_-values) and chlorophyll *a* fluorescence, providing insights into the toxicity and stress responses.

### 4.2 Preparation of Biomicroplastics and Artificial Seawater

Pristine CA (Solvay, Ocalio), PHB (Biomer, P3380), and PLA (NatureWorks LLC, Polymer 3051D) were purchased as polymer pellets; these were cryogenically milled and sieved (VS1000, Retsch, Germany) with wire sieves at 50 Hz for 10 min to obtain particles of 1–1.25 mm.

The artificial seawater, UCM, that was used for the aging experiments, was prepared as described by Califano and Wichard.^23^

### 4.3 Microplastic Degradation and Formation of Leaching Extract_1 during Aging

Photochemical aging was conducted in an aluminum-walled reactor equipped with a high-pressure UV lamp (Osram, Supratec HTT 150 211, 165 W, 230 V, irradiance of 67 mW × cm^1^ ± 10%, 280–450 nm) at a distance of 30 cm, below 30 °C (schematic structure in Figure S-1).

For the aging experiment, 45 g of PLA or PHB or 9 g of CA microplastics (1–1.25 mm) were placed in a glass vessel (⌀10 cm) with 150 mL of UCM (water column: 3.5 cm) and stirred for 350 h under continuous UV irradiation (∼262 sunny days in Vienna, 12 h/day, AM 1.5; Section S-2). Control samples (virgin) were prepared similarly but were protected from light using aluminum foil. After irradiation, the microplastics were filtered. The filtrate (UCM: Leaching Extract_1_aged_, Leaching Extract_1_virgin_) was sterile-filtered (PES filter, 0.20 μm, Sarstedt, Germany), supplemented with vitamins (solution V: 2 μL/mL)^23^ and stored at −30 °C. The residue (microplastics: Polymer_aged_, Polymer_virgin_) was rinsed with water and dried at room temperature several times.

### 4.4 Preparation of the Postirradiation Leached Substances (Leaching Extract_2)

Polymer samples with UCM (PLA and PHB: 300 mg/mL; CA: 20 mg/mL) were stored in fresh UCM for 14 days (20 °C) in order to analyze the resulting leached substances that are released after irradiation. After storage, the samples were sterile-filtered (PES filter, 0.20 μm, Sarstedt, Germany); the filtrates (Leaching Extract_2_aged_, Leaching Extract_2_virgin_) were supplemented with vitamins (solution V: 1 μL/mL)^23^ and stored at −30 °C. An overview of the samples and nomenclature is shown in Figure 5.

**Figure 5:**
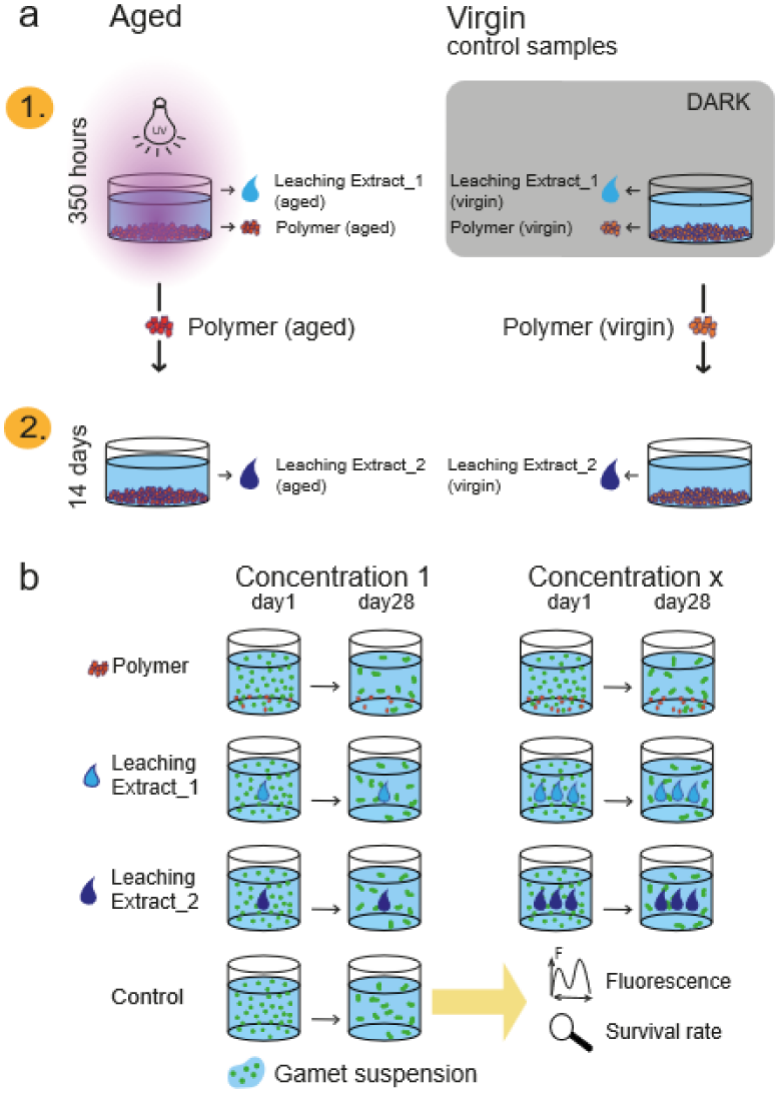
Sample preparation and bioassay performance. (a) Preparation of the aged samples (polymer, Leaching Extract_1) and the postirradiation samples (Leaching Extract_2) and their respective control samples (virgin). (b) Overview of the bioassay procedure; samples from (a) are analyzed with gametes in the bioassay and then evaluated using survival rate and chlorophyll *a* fluorescence.

### 4.5 Bioassays with *U. mutabilis*

Haploid gametes or gametophytes of *U. mutabilis* Føyn (sl-G­[mt +]; morphotype “slender”, strain FSU-UM5–1) were used. In the following, it is referred to as *U. mutabilis* (species is conspecific with *Ulva compressa*).^63^ The cultivation of this model organism was carried out in artificial seawater (UCM) under standardized conditions: 18 °C ± 2 °C, with a light/dark cycle of 17/7 h and a light intensity of 40–80 μmol photons/m^2^s^1^.^23^ Freshly discharged gametes were purified from bacteria under sterile conditions by exploiting their positive phototaxis, following the established protocol of Califano and Wichard (2018). For density control, a gamete suspension (200 gametes/μL, counted using the Neubauer improved chamber)^64^ was prepared as seed stock from axenic gametes in UCM and inoculated with *Maribacter* sp. MS6 (GenBank entry EU359911) and *Roseovarius* sp. MS2 (GenBank EU359909) (both propagated in a 50/50 marine broth and UCM).^65^ The early developmental stages of *Ulva* germination were monitored over a 14-day period and applied for multiple standardized bioassays.^21,63,65^

Different amounts of sample (Polymer_aged_, Polymer_virgin_, Leaching Extract_1_aged_, Leaching Extract_1_virgin_, Leaching Extract_2_aged_, Leaching Extract_2_virgin_) were added to 6-well plates (working volume: 4 mL). Each experimental condition was prepared in triplicate to ensure reproducibility. Tris-HCl buffer, pH 8 (56 mmol/L), was added to each well, and UCM was used to adjust the final volume to 3960 μL. Subsequently, 40 μL of the gamete suspension (final density in well: 2 gametes/μL, equal to 8000 gametes) was added while continuously swirling the suspension to counteract the phototactic movement of the gametes. In addition, control samples without microplastics were prepared in at least every second well plate, but at least six per test. The multiwell plates were incubated in the dark for 2 days and then exposed to the typical light/dark cycle.

### 4.6 Survival-Rate Analysis

Algae seedlings cultivated for 2–4 weeks were documented photographically and counted using Fiji (ImageJ). Control samples were averaged, and all values were compared with the 100% reference of the control. Concentration-effect curves were calculated in OriginPro (2023, OriginLab, USA) using a logistic function (eq 1) and the Levenberg–Marquardt algorithm (*A*_1_, lower asymptote; *A*_2_, upper asymptote; *p*, slope; *B*, inflection point). LC_50_-values were derived at *y* = 50.

Concentration-effect curve, Levenberg–Marquardt algorithm:

1 $$y=A_{1}+\frac{A_{2}-A_{1}}{1+10^{(B-x)\times p}}$$

### 4.7 Chlorophyll *a* Fluorescence Analysis

Chlorophyll *a* fluorescence of the algae seedlings (2–4 weeks cultivated) was measured with a Varioskan Flash instrument (Thermo Scientific, USA). During measurements, 797 points per well were scanned with an excitation wavelength of 430 nm and an emission wavelength of 665 nm with a 12 nm excitation bandwidth. Each well was measured three times, the values were sorted by size, and the values of the same rank were averaged. The 30 largest values, assumed to represent algal tissue, were averaged (smaller values may reflect measurements outside the algal area), and the standard error (SE) was calculated. Fluorescence values were recorded directly without background subtraction or further normalization. The approach to data processing was generally adopted from Hardegen et al.,^21^ with modifications made to account for differences in measurement conditions, well plate size, and data set.

For statistical analysis, a one-way ANOVA was performed using OriginPro software (2023, OriginLab, USA). This was performed with a Dunn–Šidák *post hoc* test; all data were compared and grouped accordingly to identify significant differences between the various equivalent concentrations (significance level: *p* < 0.05)^66^ (see details in Figure 5).

### 4.8 Material Characterization

To analyze the aging effects of the particles, comparative analyses were carried out on the virgin and aged samples using FTIR, XPS, and SEM (see Section S-3 for measurement details).

### 4.9 Molecular Fingerprint Analysis of the Leached Compounds from Aged Biopolymers (HR-MS)

The CA leaching samples (50 mL) and a UCM control (50 mL) were split into duplicates (each of 25 mL). Each replica (*n* = 10) was sterile-filtered (PES filter, 0.20 μm, Sarstedt, Germany) and subsequently loaded onto an HLB cartridge (OASIS, Waters, UK). The matrix was rinsed with 4 mL of distilled water and then eluted with methanol.^67^ The extracts were evaporated and reconstituted in 100 μL of LCMS-grade MeOH. Twenty μL of each replica and an LCMS-grade methanol blank were measured four times in negative mode, using direct inlet high-resolution Orbitrap mass spectrometry equipped with a heated electrospray source.^68^ Further details regarding instrumental setup, data processing, molecular formula assignment, and composition dissimilarity can be found in Section S-6.

### 4.10 Limitations of the Study

Several limitations should be taken into account in interpreting the results. First, the concentrations of microplastics used exceeded those typically measured in natural aquatic environments. These elevated levels were intentionally selected to ensure the detectability of potential effects and to approach LC_50_ thresholds, thereby enhancing mechanistic understanding—albeit at the expense of direct ecological transferability.

The use of artificial seawater allowed for controlled and reproducible testing, providing a simplified model to investigate microplastic toxicity. However, it does not fully replicate the complexity of natural systems, including particulate matter and diverse microbial communities, which can modulate microplastic behavior, bioavailability, and degradation dynamics. Another limitation concerns the chemical analysis of leached substances. HR-MS was used descriptively to generate a molecular fingerprint without structural identification. Many compounds likely fall outside known databases and would require targeted metabolomic or NMR analysis for further characterization—approaches beyond the scope of this exploratory study. While specific substances were not identified, the clear correlation between the number of formed compounds and observed toxicity supports the role of chemical transformation during aging.

The experiments were conducted with a single algal species, *U. mutabilis*, under laboratory conditions. While this approach ensures clarity and control, it does not capture the full ecological context in which algae interact with other organisms through competition, facilitation, or predation—factors that may influence responses in natural settings.

Nanoparticles <200 nm may have formed in the Leaching Extract_1 and Leaching Extract_2 samples and passed through the 0.2 μm filter. These were not characterized and may have contributed to the observed effects. However, their formation under the given conditions is uncertain, and their relevance remains unclear.

The microplastics were chosen for their low additive content, allowing focus on polymer-specific effects. Still, minor additive influences cannot be fully excluded and may have contributed to the observed responses. Moreover, only single commercial grades of PLA, PHB, and CA were tested. As polymer properties vary depending on synthesis routes, degrees of substitution, or residual compounds, the findings apply strictly to the specific materials investigated here. These factors should be considered when extrapolating to other grades or production processes.

Lastly, the microplastic aging process was simulated by using artificial UV exposure. This method successfully mimics key aspects of photodegradation but does not encompass the full spectrum of environmental weathering factors, such as mechanical abrasion, temperature variation, or biofilm formation. These missing factors could alter polymer degradation pathways and the chemical composition of leachates, potentially affecting the types and concentrations of substances released. As such, extrapolation to environmentally aged microplastics should be performed with caution.

## Supplementary Material



## Notes

Detailed description of polymer aging experiments and characterization methods (FTIR, XPS, SEM) with extended results, overview of all LC_50_-values and fit parameters, and instrumental setup details of HR-MS measurements (PDF)
