# Supplementary material for: The Harmful Footprint of Aged Biomicroplastics on Algal Development: A Comparative Study of Polylactic Acid, Polyhydroxybutyrate, and Cellulose Acetate
Source: ACS Omega. 2025 Oct 29;10(44):52976–85. doi: 10.1021/acsomega.5c06359 (PMC12612925; doi:10.1021/acsomega.5c06359)
Supplement: Supplementary file 1 [file ao5c06359_si_001.pdf]

Supporting Information

The harmful footprint of aged bio-microplastics on algal development: A comparative study of polylactic acid, polyhydrodybutyrate and cellulose acetate

*Paula Walz[a], Simon B. Redlich[b], Marius Hermesdorf[a], Laura Calderón-Rodríguez[c], Marcus Franke[a], Desirée Leistenschneider[a], Quirina Roode-Gutzmer[a], Felix H. Schacher[c, d], Michael Stelter[a, d, e], Thomas Wichard[b], and Patrick Braeutigam[a, d, f, g]**

[a] Friedrich Schiller University Jena Faculty of Chemistry and Earth Sciences, Institute of Technical Chemistry and Environmental Chemistry, Philosophenweg 7a, 07743 Jena, DE

[b] Friedrich Schiller University Jena Faculty of Chemistry and Earth Sciences, Institute of Inorganic and Analytical Chemistry, Lessing Str. 8, 07743 Jena, DE

[c] Friedrich Schiller University Jena Faculty of Chemistry and Earth Sciences, Institute for Organic Chemistry and Macromolecular Chemistry, Lessing Str. 8, 07743 Jena, DE

[d] Friedrich Schiller University Jena Center for Energy and Environmental Chemistry Jena, Philosophenweg 7a, 07743 Jena, DE

[e] Fraunhofer IKTS Hermsdorf, Michael-Faraday-Str. 1, Hermsdorf 07629, DE

[f] University of Stuttgart Faculty 2 Civil and Environmental Engineering, ISWA | Institute for Sanitary Engineering, Water Quality and Solid Waste Management, Bandtäle 2, 70569 Stuttgart, DE

[g] University of Stuttgart Faculty 2 Civil and Environmental Engineering, Micropollutants Competence Centre Baden-Württemberg, Bandtäle 2, 70569 Stuttgart, DE

* patrick.braeutigam@iswa.uni-stuttgart.de

Content

S-1 - UV-Reactor 3
S-2 - Lamp intensity 4
S-3 - Characterization methods 5
S-3.1 - Fourier-transform infrared spectroscopy (FTIR) 5
S-3.2 - X-ray photoelectron spectroscopy (XPS) 9
S-3.3 - Scanning Electron Microscopy (SEM) 12
S-4 - Overview LC_{50}-Value 14
S-5 - Fit parameters 15
S-6 - HR-MS Instrumental setup 16
References 19

S-1 - UV-Reactor

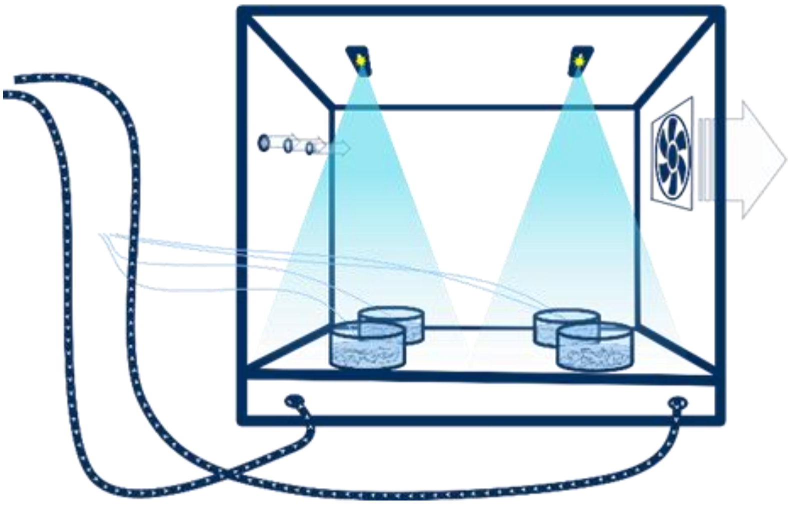

Figure S-1. Schematic representation of the UV reactor with UV lamps for the irradiation of the bio-microplastic samples in synthetic seawater.

A water-cooled floor and fan-assisted airflow maintained the chamber < 30 °C. The evaporated water was refilled every hour (ultra-pure water 18.2 MΩ·cm at 25°C ≤ 5 ppb TOC).

S-2 - Lamp intensity

Spectral irradiance – Lamp: 4173.413[W/m^{2}µm]

Spectral irradiance – Sun: 463.744 [W/m^{2}µm] [1]

$$Spectral\ irradiance\ ratio = \frac{Spectral\ irradiance_{lamp}}{Spectral\ irradiance_{sun}} = \frac{4173.413\ \frac{W}{m^2\mu m}}{463.744\ \frac{W}{m^2\mu m}} \approx 9$$

$$Time\ ratio = \frac{Operating\ time\ of\ the\ lamp\ per\ day}{Assumption\ of\ solar\ radiation\ time\ per\ day} = \frac{24}{12} = 2$$

$$\begin{aligned} &Equivalent\ time\ in\ the\ sun \\ &\qquad = Irradiation\ time \times Spectral\ irradiation\ ratio \times Time\ ratio \\ &\qquad = 350\ h \times 9 \times 2 = 6300\ hours \end{aligned}$$

The lamp used in this study emits in a range of 250-450 nm. Terrestrial solar radiation is between 305-2500 nm. In the intersection range between 305 and 450 nm, the average lamp radiation is ~9 times higher than the average solar radiation in the same range. This is based on the maximum solar altitude in Vienna. The lamp shines 24 hours a day, whereas the sun shines about 12 hours a day. This implies that 24 hours under the UV lamp correspond to about 18 days of irradiation in intense sunshine in Vienna. The 350 hours of irradiation in this experiment therefore correspond to around 262 days of intense sunshine (12 h per day) in Vienna.

S-3 - Characterization methods

S-3.1 - Fourier-transform infrared spectroscopy (FTIR)

FTIR measurements were performed using an IRSpirit FTIR spectrometer (Shimadzu, Japan) with a QATR-S diamond attachment. For each polymer sample (PLA_{aged}, PLA_{virgin}, PHB_{aged}, PHB_{virgin}, CA_{aged}, CA_{virgin}), three measurements were taken on different particles for representativity. Spectra were acquired from 45 scans in transmission mode in the wavenumber range of 4000 to 500 cm^{-1} at a resolution of 16 cm^{-1} (Figure S-2). Characteristic peaks are tabulate in Table S-1 and shown in Figure S-2. A background spectrum was subtracted before each measurement, and the three spectra per particle were averaged.

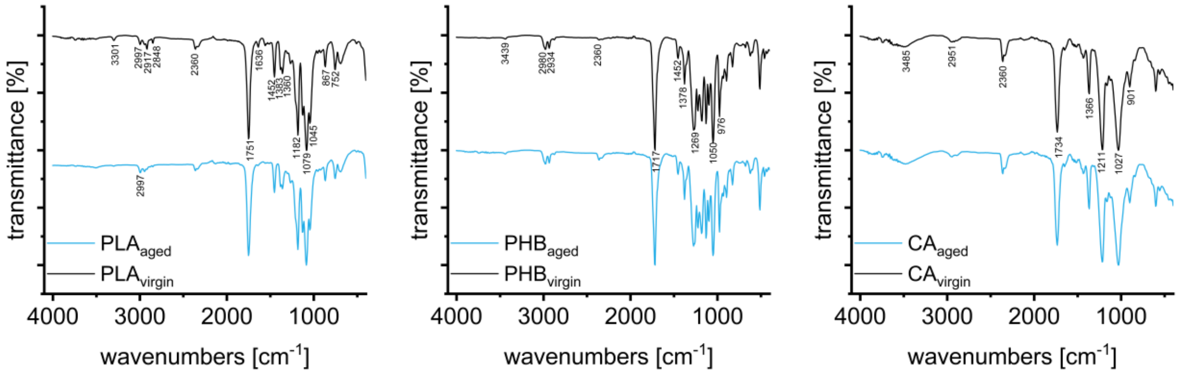

Figure S-2. FTIR spectra of the aged microplastic samples PLA_{aged}, PHB_{aged}, CA_{aged} (350 h UV irradiation in artificial seawater) with the control samples PLA_{virgin}, PHB_{virgin}, CA_{virgin} (350 h in artificial seawater without UV irradiation).

Table S-1. Characteristic wavenumbers [cm^{-1}] of the FTIR measurement with associated bands and their assignment of PLA[2]

**Wavenumber (cm^{-1})**	**Bond / Chemical Groups**
3301	OH stretching vibration (possible degradation products like lactic acid or oligomers) (PLA_{virgin})
2997, 2948	Asymmetric and symmetric CH_3 stretching vibrations
2360	CO_2 absorption (From ambient air. More pronounced in aged samples)
1751	C=O stretching (ester group), strong absorption band characteristic of PLA
1636	H-O-H bending vibration, Possible water residues (more intense in PLA_{virgin})
1452, 1383, 1360	CH_3 asymmetric and symmetric bending
1182, 1125, 1079, 1045	C-O stretching vibrations
867, 752	C-COO bending vibration, C-H group

Table S-2. Characteristic wavenumbers [cm-1] of the FTIR measurement with associated bands and their assignment of PHB[3-6]

**Wavenumber (cm^{-1})**	**Bond / Chemical Groups**
3439	O-H stretching (terminal hydroxyl groups)
2980, 2934	Alkyl CH_3 group
2360	CO_2 in the environment (stronger in PHB_{aged})
1717	C=O stretching vibration (ester group)
1452	Asymmetric CH_2 or CH_3 bending
1378	Symmetric CH_3 bending
1300-1000	C-O stretching vibrations, C-O-C stretching vibrations (ester groups)

Table S-3. Characteristic wavenumbers [cm^{-1}] of the FTIR measurement with associated bands and their assignment of CA[7]

**Wavenumber [cm^{-1}]**	**Bond / Chemical Groups**
2951	Asymmetric C-H stretching vibration (methyl groups)
2360	CO_2 asymmetric stretching absorption (ambient contamination)
1734	C=O stretching vibration (ester groups)
1366	C-H deformation vibration (methyl groups)
1211	C-O stretching vibration (ester bonds)
1027	C-O stretching vibration (alcohol groups) or C-O vibrations (acetyl ester groups)
901	C-O-C bending vibration (glycosidic bond in the cellulose backbone)

S-3.2 - X-ray photoelectron spectroscopy (XPS)

XPS measurements were conducted using a K-alpha spectrometer (Thermo Fisher Scientific, USA). The samples were fixed with an adhesive Cu-foil onto the sample holder. A flood gun, calibrated to C=C in PE at 284.8 eV ± 0.1 eV, was used for charge compensation. The pressure in the measurement chamber was below $4\cdot10^{-7}$ mbar. The recorded spot size was 300 µm. For compositional data, survey spectra were recorded with a 1 eV step size and a pass energy of 100 eV. High-resolution spectra were acquired using a 0.05 eV step size and a pass energy of 30 eV, with each 5 scans for C1s and O1s. All high-resolution spectra were analyzed using Avantage v6.6.0 software from Thermo Fisher Scientific.

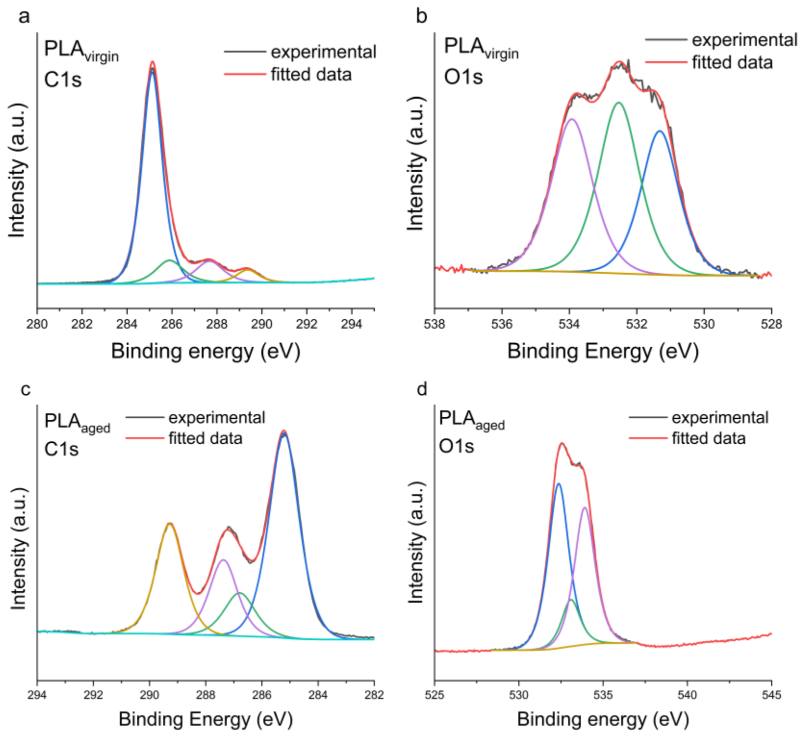

Figure S-3: X-ray photoelectron spectra of (a&b) PLA_{virgin} (without UV irradiation) and (c&d) PLA_{aged} (350 h UV irradiation).

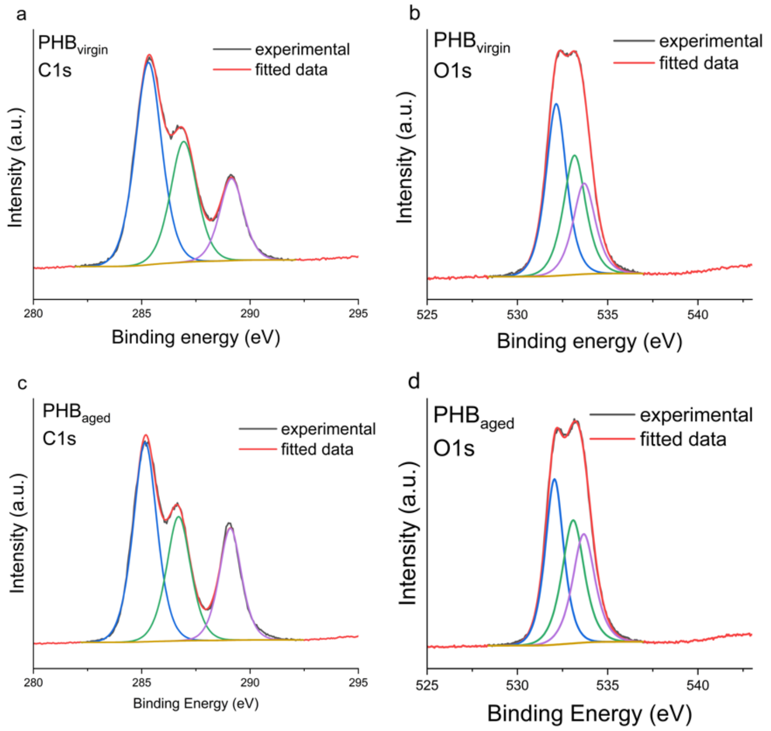

Figure S-4. X-ray photoelectron spectra of (a&b) PHB_{virgin} (without UV irradiation) and (c&d) PHB_{aged} (350 h UV irradiation).

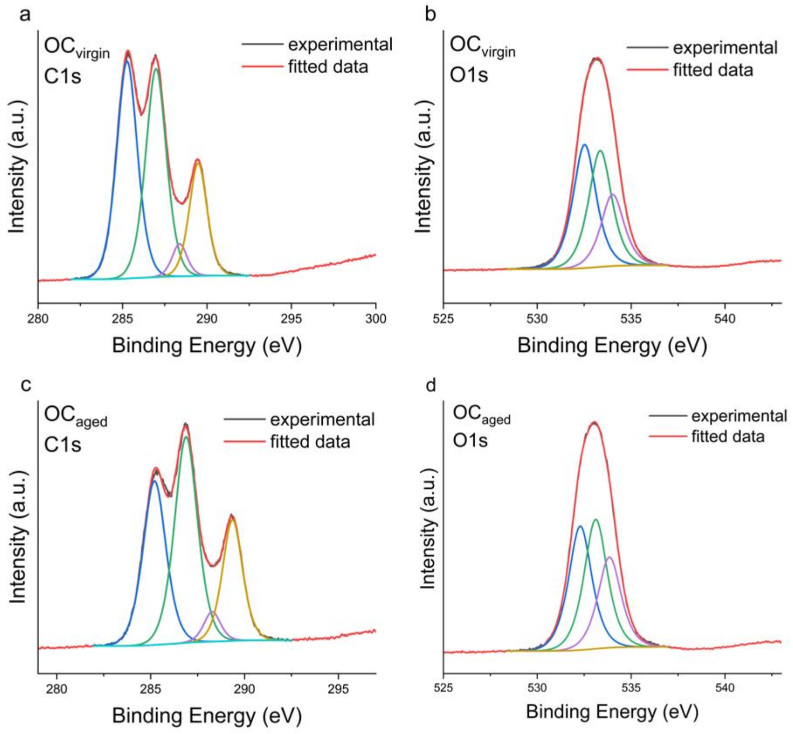

Figure S-5. X-ray photoelectron spectra: (a&b) CA$_{virgin}$ (without UV irradiation) and (c&d) CA$_{aged}$ (350 h UV irradiation).

S-3.3 - Scanning Electron Microscopy (SEM)

A Sigma VP Field Emission SEM (Carl-Zeiss AG, Germany) was used for imaging. Samples were fixed on carbon adhesive discs, coated with Pt (5 nm) in a high-vacuum sputter coater (Safematic CCU-O10 HV) to prevent charging, and a small amount of conductive silver paint was applied for better conductivity. Micrographs were captured at 4 kV acceleration voltage and 258-271 pA emission current using an SE2 detector.

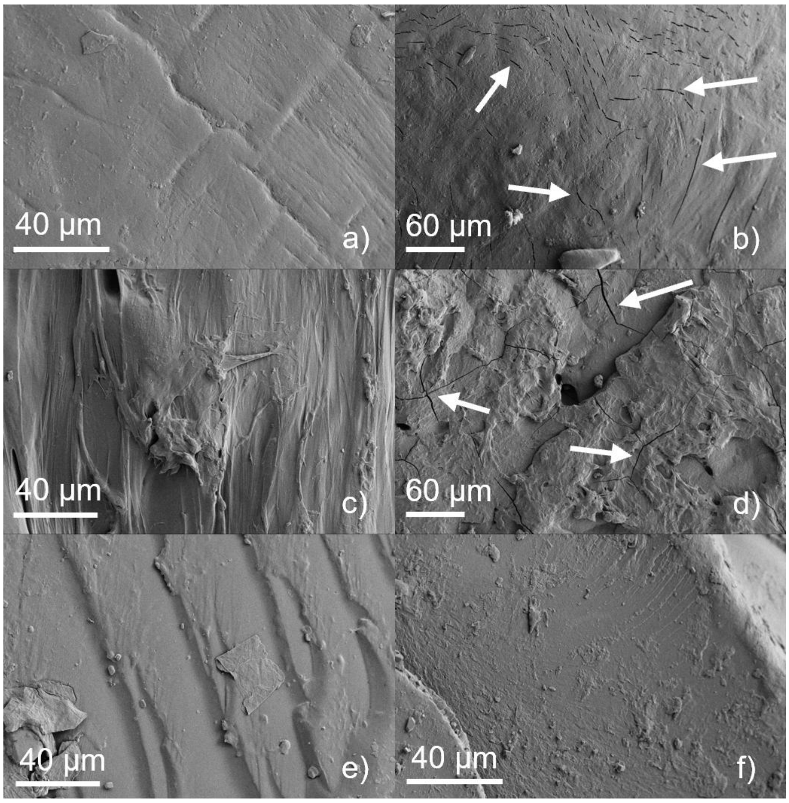

Figure S-6. SEM images comparing aged microplastic samples b) PLA_{aged} d) PHB_{aged} f) CA_{aged} (350 h UV irradiation in artificial seawater) with control samples a) PLA_{virgin} c) PHB_{virgin} e) CA_{virgin} (350 h in artificial seawater without UV-irradiation); arrows indicates cracks.

The non-aged and aged polymer samples of each bio-microplastic, following 350 h of photochemical treatment, were compared using surface characterization methods. Surface changes, such as increased roughness, due to weathering is a classic feature of polymer ageing.[6, 8-11] Four particles at different magnifications were analyzed using SEM. No cracks were detected in any virgin particles. The most significant effects from UV irradiation were observed in PLA_{aged}, which showed surface cracks in 3 out

of 4 particles. For PHB_{aged}, cracks could only be detected in one of four particles, and no cracks were detected in CA_{aged}. The differences in roughness was challenging to determine, as they varied according to manufacturing process; extruded surfaces were smooth, while cut surfaces were rougher.

S-4 - Overview LC_{50}-Value

Table S-4. Summary of LC_{50}-values for aged and virgin samples of the three microplastic types (1-1.25 mm), Leaching Extract_1 and Leaching Extract_2 (PLA, PHB, CA).

	**Aged [mg/mL]**	**Virgin [mg/mL]**
**PLA-Polymer**	35.08	158.03
**Leaching Extract_1$_{PLA}$**	37.04	>250
**Leaching Extract_2$_{PLA}$**	82.52	>250
**PHB-Polymer**	16.35	175.49
**Leaching Extract_1$_{PHB}$**	46.35	>250
**Leaching Extract_2$_{PHB}$**	204.10	>250
**CA-Polymer**	1.35	5.77
**Leaching Extract_1$_{CA}$**	<0.5	1.62
**Leaching Extract_2$_{CA}$**	1.12	2.41

S-5 - Fit parameters

Table S-5. Fitting parameters of the toxicity of the polymers, Leaching Extract_1 and Leaching Extract_2 of the three bio-microplastics PLA, PHB and CA; bottom asymptote was set to 0 and the top asymptote was set to 100.

	**PLA_{aged}**	**PLA_{virgin}**	**Leaching Extract_1$_{PLA,virgin}$**	**Leaching extract_1$_{PLA,aged}$**	**Leaching Extract_2$_{PLA,virgin}$**	**Leaching Extract_2$_{PLA,aged}$**
**Inflection point [mg/mL]**	1,545	2,199	2.407	1.569	-5.056	1.91
**Hill slope [mL/mg]**	-1,589	-3,822	-34.083	-2.187	0.0698	-1.072
	**PHB_{aged}**	**PHB_{virgin}**	**Leaching Extract_1$_{PHB,virgin}$**	**Leaching Extract_1$_{PHB,aged}$**	**Leaching Extract_2$_{PHB,virgin}$**	**Leaching Extract_2$_{PHB,aged}$**
**Inflection point [mg/mL]**	1.214	2.245	2.62	1.672	114.931	2.31
**Hill slope [mL/mg]**	-1.013	-0.952	-0.716	-2.111	-0.009	-1.519
	**CA_{aged}**	**CA_{virgin}**	**Leaching Extract_1$_{CA,virgin}$**	**Leaching Extract_1$_{CA,aged}$**	**Leaching Extract_2$_{CA,virgin}$**	**Leaching Extract_2$_{CA,aged}$**
**Inflection point [mg/mL]**	0,134	0,762	0.211	-0.654	0.382	0.051
**Hill slope [mL/mg]**	-1,667	-5,675	-1.060	-1.905	-0.969	-2.085

S-6 - HR-MS Instrumental setup

High resolution (Orbitrap) measurements were executed on a QExactive plus Thermo (Bremen, Germany) mass spectrometer equipped with a heated electrospray source (HESI) and coupled to a liquid chromatography system UltiMate HPG-3400 RS binary pump, a WPS-3000 auto sampler which was set to 10 °C and which was equipped with a 25 µL injection syringe and a 100 µL sample loop. Instead of a column a 0.18 mm, 600 mm length capillary was installed within the column compartment TCC-3200. For automated direct injection 20.0 µL sample was injected using the flow gradient in Table S-6 with LCMS grade Methanol. The flow was switched according to Table S-6 from waste to the MS and back to the waste, to prevent source contamination. For monitoring full scan mode was selected with the following parameters: negative polarity; scan range: 100 to 900 m/z; resolution: 280,000 (optimized for 200 m/z); AGC target: 3 × 106; maximum IT: 256 ms. General settings: sheath gas flow rate: 15; auxiliary gas flow rate 5; sweep gas flow rate: 0 arbitrary units; spray voltage: 3.5 kV; capillary temperature: 250 °C; S-lens RF level: 90; auxiliary gas heater temperature: 113 °C.

Table S-6. Flow gradient for automated direct injection

Time	Flow [mL/min]	MS Aquisition	Waste valve
0	0.1	off	waste
0.5	0.1	on	waste → MS
0.6	0.02	on	MS
4	0.02	on	MS → waste
4.1	1	off	waste
5	1	off	waste
5.1	0.2	off	waste
5.5		off	waste → MS
5.8		off	MS → waste
6	0.2	off	waste

Data processing

Raw data files were converted to the mzML format using MSConvert (ProteoWizard v3.0.2).[12] An average mass spectrum was generated by merging 20 consecutive scans (scans 80–119) using a custom R script based on the XCMS and MSnbase packages.[13, 14] To reduce spectral noise, only signals detected in at least 4 out of the 20 scans (≥20%) within a mass deviation threshold of 1.5 ppm reducing noise. A linear internal mass recalibration was performed using OpenMS (v3.3.0), based on a molecular formula list proposed by Hawkes et al. (2020).[15] A feature list is subsequently generated by OpenMS and converted into a file format compatible with the browser-based UltraMassExplorer (UME) version 1.1.0 for molecular formula assignment.

Molecular Formula assignment

Molecular formulas were assigned with the browser-based UltraMassExplorer (UME) v 1.1.0.[16] The sum formula calculations considered the following isotopes: 12C (0 - ∞), 1H (0 - ∞), 16O (0 - ∞), 14N (0 - 3), 32S (0 - 1), 31P (0 - 1). The analytical error was maintained below 3 ppm. The nitrogen rule was applied automatically, elemental ratio thresholds were set at $O/C \leq 1.1$ and $0.3 \leq H/C \leq 2.3$ and double bond equivalent minus oxygen threshold to $-10 < \text{DBE-O} < 10$.[17, 18] To consider an assigned formular correct the 13C-isotope signal had to be present (verification of the parent ion) but were subsequently removed from the final dataset. Formulas detected in the any of the methanol blank and UCM control measurements were removed from each measurement of leaching samples. It is important to note that some signals can have multiple molecular formulae assigned. To choose the right molecular formula following rules with priority as follows were implemented: (1) minimum number of N+S+P; (2) minimum number of S+P; (3) lowest error. The peak intensities were normalized to the sum of intensities of all identified molecular formulas of one sample.[19]

Composition dissimilarity

To enable robust statistical analysis, replicate measurements were condensed by retaining only those signals present in all four technical replicates. For each retained signal, the m/z values and intensities were averaged to generate a representative feature list per condensed replicate. These feature lists were subsequently processed to the same molecular formula assignment criteria as described previously. Based on the resulting normalized intensities, UltraMassExplorer (UME) performed a Bray-Curtis dissimilarity clustering analysis[20] across all condensed replicates.

% Bray-Curtis dissimilarity calculation (1): signal intensity I is compared between sample p and q for each molecular mass k (form k1 to kn)

$$\%BC\ dissimilarity = 100\frac{\sum_{k=1}^{n}\left|I_{p,k} - I_{q,k}\right|}{\sum_{k=1}^{n}\left|I_{p,k} + I_{q,k}\right|} \quad (1)$$

References

(1) Rottman, G. Measurement of Total and Spectral Solar Irradiance. *Space Science Reviews* **2007**, *125* (1-4), 39-51. DOI: 10.1007/s11214-006-9045-6.

(2) Tamayo-Belda, M.; Venâncio, C.; Fernandez-Piñas, F.; Rosal, R.; Lopes, I.; Oliveira, M. Effects of petroleum-based and biopolymer-based nanoplastics on aquatic organisms: A case study with mechanically degraded pristine polymers. *Science of The Total Environment* **2023**, *883*, 163447. DOI: https://doi.org/10.1016/j.scitotenv.2023.163447.

(3) Ramezani, M.; Amoozegar, M. A.; Ventosa, A. Screening and comparative assay of poly-hydroxyalkanoates produced by bacteria isolated from the Gavkhooni Wetland in Iran and evaluation of poly-β-hydroxybutyrate production by halotolerant bacterium Oceanimonas sp. GK1. *Annals of Microbiology* **2015**, *65* (1), 517-526. DOI: 10.1007/s13213-014-0887-y.

(4) Trakunjae, C.; Boondaeng, A.; Apiwatanapiwat, W.; Kosugi, A.; Arai, T.; Sudesh, K.; Vaithanomsat, P. Enhanced polyhydroxybutyrate (PHB) production by newly isolated rare actinomycetes Rhodococcus sp. strain BSRT1-1 using response surface methodology. *Scientific Reports* **2021**, *11* (1), 1896. DOI: 10.1038/s41598-021-81386-2.

(5) Saad, G. R.; Khalil, T. M.; Sabaa, M. W. Photo- and bio-degradation of poly(ester-urethane)s films based on poly[(R)-3-hydroxybutyrate] and poly($\mathcal{E}$-caprolactone) blocks. *J Polym Res* **2010**, *17* (1), 33-42, Article. DOI: 10.1007/s10965-009-9287-6 Scopus.

(6) Sadi, R. K.; Fechine, G. J. M.; Demarquette, N. R. Photodegradation of poly(3-hydroxybutyrate). *Polymer Degradation and Stability* **2010**, *95* (12), 2318-2327. DOI: https://doi.org/10.1016/j.polymdegradstab.2010.09.003.

(7) Arundati, A. H.; Ratri, C. R.; Chalid, M.; Aqoma, H.; Nugraha, A. F. A combination of nonsolvent and thermally induced phase separation (N-TIPS) technique for the preparation of highly porous cellulose acetate membrane as lithium-ion battery separators. *Ionics* **2024**, *30* (1), 123-133. DOI: 10.1007/s11581-023-05276-5.

(8) Abaroa-Pérez, B.; Ortiz-Montosa, S.; Hernández-Brito, J. J.; Vega-Moreno, D. Yellowing, Weathering and Degradation of Marine Pellets and Their Influence on the Adsorption of Chemical Pollutants. *Polymers* **2022**, *14* (7), 1305. DOI: 10.3390/polym14071305.

(9) Sun, J.; Zheng, H.; Xiang, H.; Fan, J.; Jiang, H. The surface degradation and release of microplastics from plastic films studied by UV radiation and mechanical abrasion. *Science of The Total Environment* **2022**, *838*, 156369. DOI: https://doi.org/10.1016/j.scitotenv.2022.156369.

(10) Xiu, H.; Qi, X.; Bai, H.; Zhang, Q.; Fu, Q. Simultaneously improving toughness and UV-resistance of polylactide/titanium dioxide nanocomposites by adding poly(ether)urethane. *Polymer Degradation and Stability* **2017**, *143*, 136-144. DOI: https://doi.org/10.1016/j.polymdegradstab.2017.07.002.

(11) Man, C.; Zhang, C.; Liu, Y.; Wang, W.; Ren, W.; Jiang, L.; Reisdorffer, F.; Nguyen, T. P.; Dan, Y. Poly (lactic acid)/titanium dioxide composites: Preparation and performance under ultraviolet irradiation. *Polymer Degradation and Stability* **2012**, *97* (6), 856-862. DOI: https://doi.org/10.1016/j.polymdegradstab.2012.03.039.

(12) Chambers, M. C.; Maclean, B.; Burke, R.; Amodei, D.; Ruderman, D. L.; Neumann, S.; Gatto, L.; Fischer, B.; Pratt, B.; Egertson, J.; et al. A cross-platform toolkit for mass spectrometry and proteomics. *Nature Biotechnology* **2012**, *30* (10), 918-920. DOI: 10.1038/nbt.2377.

(13) Gatto, L.; Lilley, K. S. MSnbase-an R/Bioconductor package for isobaric tagged mass spectrometry data visualization, processing and quantitation. *Bioinformatics* **2012**, *28* (2), 288-289. DOI: 10.1093/bioinformatics/btr645.

(14) Smith, C. A.; Want, E. J.; O'Maille, G.; Abagyan, R.; Siuzdak, G. XCMS: Processing Mass Spectrometry Data for Metabolite Profiling Using Nonlinear Peak Alignment, Matching, and Identification. *Analytical Chemistry* **2006**, *78* (3), 779-787. DOI: 10.1021/ac051437y.

(15) Hawkes, J. A.; D'Andrilli, J.; Agar, J. N.; Barrow, M. P.; Berg, S. M.; Catalán, N.; Chen, H.; Chu, R. K.; Cole, R. B.; Dittmar, T.; et al. An international laboratory comparison of dissolved organic matter composition by high resolution mass spectrometry: Are we getting the same answer? *Limnology and Oceanography: Methods* **2020**, *18* (6), 235-258. DOI: 10.1002/lom3.10364.

(16) Leefmann, T.; Frickenhaus, S.; Koch, B. P. UltraMassExplorer: a browser-based application for the evaluation of high-resolution mass spectrometric data. *Rapid Communications in Mass Spectrometry* **2019**, *33* (2), 193-202. DOI: 10.1002/rcm.8315.

(17) Castro-Morales, K.; Canning, A.; Arzberger, S.; Sellmaier, S.; Redlich, S.; Overholt, W. A.; Zimov, N.; Marca, A.; Kaiser, J.; Wichard, T.; et al. Using O2/Ar ratios as a proxy for biological productivity determinations in an Arctic river. Copernicus GmbH: 2022.

(18) Herzsprung, P.; Hertkorn, N.; Von Tümpling, W.; Harir, M.; Friese, K.; Schmitt-Kopplin, P. Molecular formula assignment for dissolved organic matter (DOM) using high-field FT-ICR-MS: chemical perspective and validation of sulphur-rich organic components (CHOS) in pit lake samples. *Analytical and Bioanalytical Chemistry* **2016**, *408* (10), 2461-2469. DOI: 10.1007/s00216-016-9341-2.

(19) Fu, Q.-L.; Fujii, M.; Riedel, T. Development and comparison of formula assignment algorithms for ultrahigh-resolution mass spectra of natural organic matter. *Analytica Chimica Acta* **2020**, *1125*, 247-257. DOI: 10.1016/j.aca.2020.05.048.

(20) Bray, J. R.; Curtis, J. T. An Ordination of the Upland Forest Communities of Southern Wisconsin. *Ecological Monographs* **1957**, *27* (4), 325-349. DOI: 10.2307/1942268.
